# Cbl upregulates *cysH* for hydrogen sulfide production in *Aeromonas veronii*

**DOI:** 10.7717/peerj.12058

**Published:** 2021-09-09

**Authors:** Yidong Zhang, Zebin Liu, Yanqiong Tang, Xiang Ma, Hongqian Tang, Hong Li, Zhu Liu

**Affiliations:** Hainan University, Haikou, China

**Keywords:** SmpB, Cbl, H_2_S metabolism, Regulation

## Abstract

Endogenous hydrogen sulfide (H_2_S) is generated in many metabolism pathways, and has been recognized as a second messenger against antibiotics and reactive oxygen species (ROS). In *Aeromonas veronii*, Small Protein B (SmpB) plays an important role in resisting stress. The absence of *smpB* could trigger sulfate assimilation pathway to adapt the nutrient deficiency, of which was mediated by up-regulation of *cbl* and *cys* genes and followed with enhancing H_2_S production. To figure out the mutual regulations of *cbl* and *cys* genes, a series of experiments were performed. Compared with the wild type, *cysH* was down-regulated significantly in *cbl* deletion by qRT-PCR. The fluorescence analysis further manifested that Cbl had a positive regulatory effect on the promoter of *cysJIH*. Bacterial one-hybrid analysis and electrophoretic mobility shift assay (EMSA) verified that Cbl bound with the promoter of *cysJIH*. Collectively, the tolerance to adversity could be maintained by the production of H_2_S when SmpB was malfunctioned, of which the activity of *cysJIH* promoter was positively regulated by upstream Cbl protein. The outcomes also suggested the enormous potentials of *Aeromonas veronii* in environmental adaptability.

## Introduction

*Aeromonas veronii* is widely present in fresh water, sewage, soil and even sea water (Hickman-Brenner et al., 1987), which endows with strong resistance to multiple antibiotics (Liu et al., 2018). Small Protein B (SmpB) acts as a small RNA binding protein in the trans-translation system to help transfer messenger RNA (tmRNA) to rescue the retained ribosomes in bacteria (Wali Karzai, Susskind & Sauer, 1999). Also, SmpB performs many significant functions in biological regulation. For example, the expression of ribonuclease R (RNase R), an exonuclease molecule that recognizes and degrades RNA, depends on SmpB in *Streptococcus pneumoniae* (Moreira et al., 2012). SmpB protein promotes the binding and degradation of RNase R by HslUV and Lon in *Escherichia coli* (Liang & Deutscher, 2012). Moreover, SmpB has similar effects with the known RNA chaperone proteins such as CrsA and Hfq. The loss of SmpB affects 4% transcription changes of genes in *salmonella*, of which involves in biological processes, including invasion, bacterial movement, central metabolism, lipopolysaccharide (LPS) biosynthesis, two-component regulatory system and fatty acid metabolism (Sittka et al., 2007; Ansong et al., 2009). In all, SmpB is essential for intra-macrophage proliferation and the strong adaptability to oxidative stress in *Salmonella* (Ansong et al., 2009). However, the mechanism of SmpB increasing the adaptability to stress is vague. To survive in oxidative damage, the bacteria evolve the enzymatic antioxidants such as superoxide dismutase (SOD) to tolerate peroxide anion radicals (He et al., 2017). Furthermore, bacterial hydrogen sulfide (H_2_S) could increase SOD activity and maintain redox balance in *vivo* (Shukla et al., 2017). H_2_S is a gaseous molecule with an unpleasant smell which at high concentrations is toxic to most living organisms/live cells, etc. (Lindenmann et al., 2010). However, the low concentration of H_2_S participates in bacterial defense against reactive oxygen species (ROS) and antibiotics-induced oxidative damage (Lindenmann et al., 2010). Since H_2_S is water-soluble, a considerable amount of H_2_S exists in the form of HS^−1^. It is hardly distinguished whether H_2_S or HS^−1^ that contributes to the biological activity. Therefore, H_2_S mentioned in biologically relevant article virtually contains both species in case of confusion (Powell, Dillon & Matson, 2018). One of the basic H_2_S synthesis includes the sulfate assimilation pathway, which is catalyzed by *cysNDC* and *cysJIH* (Shatalin et al., 2011; Kimura, 2014; Wu et al., 2015). In *Salmonella typhimurium*, the expression of *cysJIH* is regulated by CysB which has 41% amino acid sequence homology with Cbl (Iwanicka-Nowicka et al., 2007; Álvarez et al., 2015), there is little evidence regarding the role of Cbl in regulating *cysJIH* in *Aeromonas* species. Both CysB and Cbl are LysR-type transcriptional activator. In sulfur metabolism, Cbl acts as a sensor of the intracellular sulphate level, and activates *tau* and *ssu* promoter *in vivo* and *in vitro* (Van Der Ploeg et al., 1999; Van Der Ploeg, Eichhorn & Leisinger, 2001; Bykowski et al., 2002). In addition, Cbl activates sulfate starvation-induced genes under sulfate starvation (Van Der Ploeg et al., 1999). Taking together, there may be a potential connection between Cbl and *cys* genes in the sulfate assimilation pathway.

In *Aeromonas veronii*, both SmpB and H_2_S play important roles in adverse stress. The absence of SmpB induced the generation of H_2_S helping to survive. The transcriptomic analysis revealed that both *cbl* and *cys* genes were up-regulated in SmpB deletion strain. To clarify the regulatory relationship between *cbl* and *cys* genes in the sulfate assimilation pathway, real-time PCR experiment and fluorescence analysis were performed, showing that Cbl positively regulated *cysH* gene. Furthermore, bacterial one-hybrid system and EMSA verified that Cbl regulated *cysH* by binding to the promoter of *cysJIH*. In brief, Cbl bound and activated *cysJIH* promoter directly to increase H_2_S production, remedying the survival ability after *smpB* deficiency. Our study elucidated the strong vitality and adaptability of *Aeromonas veronii* in adverse stress. We also uncovered a novel model of H_2_S biosynthetic mechanism that may be contributed to stressful survival and recalcitrance of bacterial infections.

## Materials & methods

### Bacterial strains, plasmids and culture conditions

The bacterial strains and plasmids used in this study were shown in Table S1. The *smpB* deletion strain of *Aeromonas veronii* C4 was constructed previously (Liu et al., 2015). The derivative *Aeromonas veronii* C4 strains were grown in LB/M9 medium supplemented with 50 mg/mL ampicillin at 30 °C, and *E. coli* strains were grown in LB medium supplemented with 50 mg/mL kanamycin and 25 mg/mL chloramphenicol at 37 °C. And all plasmids were sequenced for verification. LB medium contained 10% tryptone, 5% yeast extract and 10% NaCl. M9 medium contained 20% 5×M9 salts, 0.2% 1 M MgSO_4_, 0.01% 1 M CaCl_2_ and 0.4% Glucose, of which 5×M9 salts included 6.4% Na_2_PO_4_·7H_2_O, 1.5% KH_2_PO_4_, 0.25% NaCl and 0.5% NH_4_Cl.

### Complemented strain construction

The DNA fragment including both *cbl* gene and its promoter was amplified by PCR (Wang et al., 2019). PCR reaction was performed as follow: 98 °C for 2 min, stepped by 98 °C for 30 s, 55 °C for 30 s and 72 °C for one kb/min in 30 cycles. The purified PCR product was inserted into pBBR plasmid for creating pBBR-Cbl expression plasmid. The *cbl* deletion strains were complemented by the conjugation of recipient WM3064 strains carrying pBBR-Cbl.

### H_2_S detection

The Pb(Ac)_2_ detection (Shatalin et al., 2011) method and WSP5 fluorescent H_2_S probe (Peng et al., 2014) were performed for monitoring H_2_S production in gas and liquid phases, respectively. Bacteria were grown in M9 at 30 °C for 48 h with soaked Pb(Ac)_2_ paper strips hanging from the mouth of the conical bottle, and five mg/L Na_2_SO_3_ was added as a source of sulfur. Pb(Ac)_2_-soaked paper strips showed a PbS brown stain as a result of the reaction with H_2_S. The color length of one mm represented 12 μg/L of H_2_S production. After 10 μM WSP5 was added to liquid bacterial culture, the samples were incubated at 37 °C for 30 min and then washed in PBS buffer to remove excess probe. Synergy H1 (BioTek) was used to take fluorescent readings at excitation 500 nm and emission 533 nm. Each reaction was performed at least in triplicate.

### RNA extraction and qRT-PCR

The qPCR reaction was conducted with ABI Prism^®^ 7300 (ABI, New York, NY, USA) for fluorescent detection utilizing SYBRR^®^ Green real time PCR Master Mix (Toyonbo, Shanghai, China). The cDNA was synthesized by RNA reverse transcription reaction and was used as the template for real-time PCR. The primers used to monitor expression of the objective genes were summarized in Table S2. Each reaction was performed at least in triplicate, and wild type (WT) was chosen as the control. And the data was analyzed by the comparative CT method (Schmittgen & Livak, 2008).

### Fluorescence analysis

The promoter of *cysJIH* was fused with eGFP and inserted into pUC19 plasmid. The *cbl* gene was cloned into pTRG plasmid simultaneously. Both the above plasmids were co-transformed into *E. coli* Reporter strain. Meanwhile, the recombinant pUC19 plasmid and the empty pTRG plasmid were co-transformed as the negative control. After bacteria were grown in LB at 37 °C, the total amount of 1 × 10^8^ cells were harvested in 1.5 mL eppendorf tube at interval time. The samples were washed with PBS twice, and placed on Synergy H1 (Biotek) for the fluorescent readings at excitation 425 nm and emission 525 nm. Each reaction was performed at least in triplicate.

### Bacterial one-hybrid analysis

To identify whether the transcription factor Cbl interacted with the promoter of *cysJIH*, Cbl was inserted into pTRG, and the promoter of *cysJIH* was ligated with pBXcmT, following with both the recombinant plasmids were cotransformed into *E. coli* Reporter strain. The transformants were placed on a selective NM medium plate containing five mM 3-amino-1, 2, 4-triazole (3-AT) and 12.5 mg/mL streptomycin for incubation at 37 °C for 48 h. The pTRG and pBXcmT plasmids were co-transformed as the negative control, and pTRG-GAL and pBT-LGF2 were co-transformed as the positive control.

### Protein expression and purification

The *cbl* gene was inserted into pET28a plasmid and transformed into *E. coli* BL21 strain. The expression and purification were performed according to previous procedure (Bykowski et al., 2002). Cbl protein was purified from *E. coli* BL21 harboring the pET28a-Cbl plasmid. The recombinant bacteria were grown in LB at 37 °C until the logarithmic phase, followed by the addition of 0.1 mM IPTG to induce protein expression at 15 °C for 14 h. The cells were harvested in Tris-HCl and lysed by sonication. The supernatant was collected after centrifugation and loaded onto a Ni-NTA column. The sample was eluted prior to the dialysis, and SDS-PAGE was used to assess protein purity.

### Electrophoretic mobility shift assay (EMSA)

Double stranded DNA probes were radiolabeled with Fluorophore 6-carboxy-fluorescein (FAM) and purified by FastPure Gel DNA Extraction Mini Kit (Vazyme). For the EMSA, DNA probe was incubated with Cbl protein samples in reaction buffer (10 mM Tris–HCl, one mM MgCl_2_, one mM DTT, 40 mM KCl, 0.1 mg/mL BSA, 5% (w/v) glycerol) at 37 °C for 30 min. After the samples were separated using a 6% native acrylamide gel (Zhang et al., 2020), the gel was then exposed to a phosphorscreen and visualized on Typhoon FLA 9500.

### Transcriptome analysis

To perform the whole-transcriptome analysis, the wild type and *smpB* deletion of *Aeromonas veronii* C4 were grown in M9 at 30 °C for 20 h, and 2 OD_600_ of cells were collected. Illumina HiSeq-X ten based on the service of RNA-Seq Quantification library at BGI-Shenzhen (China) was used to obtain the transcriptome sequencings. And the RNA-seq raw data was assembled and analyzed by comparing with the translational region of the annotated DNA sequence in reference genome (GCA_001593245.1) using HISAT (Kim, Langmead & Salzberg, 2015). The DESeq. 2 package in R was used for the estimation of fold changes and other analysis (Love, Huber & Anders, 2014).

### Statistical analyses

Statistical significance was determined by t test (two-tailed distribution with two-sample, equal variance) when directly comparing two conditions or a one-way analysis of variance (ANOVA) and Tukey post-test by pairwise comparisons.

## Results

### Transcriptomic analysis

**Figure 1:**
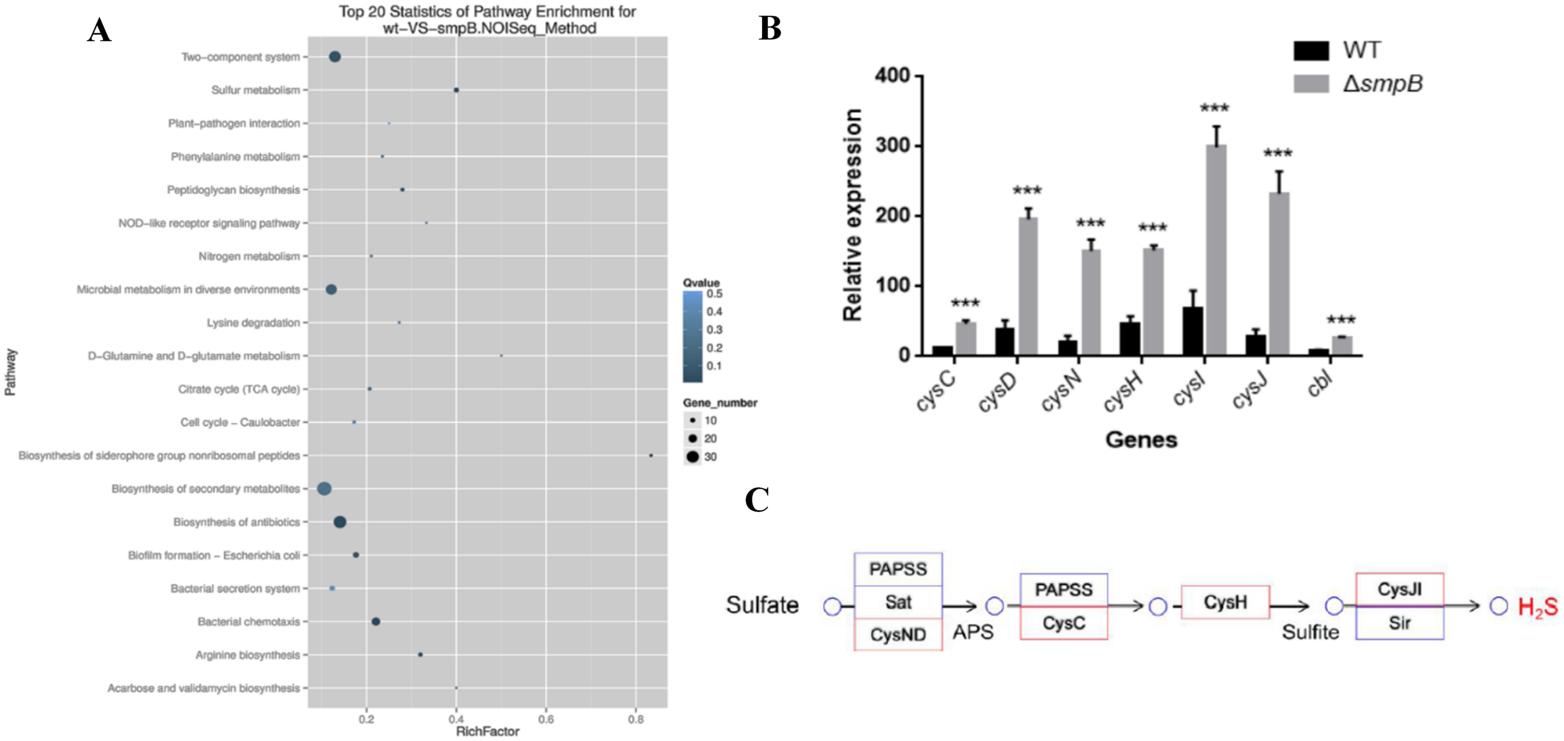
Transcriptomic analysis between wild type (WT) and *smpB* knockout. (A) The KEGG pathways for the different metabolites between WT and the *smpB* deletion (Δ*smpB*). (B) The relative expression of the correlated H_2_S synthesis genes in WT and Δ*smpB* cells. Values represented means ± SD (*n* = 3). ***p < 0.001 was determined by one-way ANOVA and Tukey post-test. (C) The deletion of *smpB* enhanced the expression of genes in the sulfate assimilation pathway.

Based on the transcriptomic analysis, the deletion of SmpB mainly caused the changes in 20 biological pathways, including two-component system, sulfur metabolism, plant pathogenic bacteria interaction, and phenylalanine metabolism. Sulfur metabolism was the most influential on metabolic pathways (Fig. 1A).

In *Aeromonas veronii* C4, the H_2_S synthesis pathway included the sulfate assimilation pathway, the organic pathway, and the 3-MST pathway. But compared with others, *Aeromonas veronii* C4 lacked cystathionine β-synthase (CBS) in the transsulfuration pathway and cysteine aminotranferase (CAT) in the 3MST pathway. The deletion of SmpB mainly up-regulated the transcription levels of *cysN*, *cysD*, *cysC*, *cysH*, *cysJ*, *cysI* and *cbl* (Fig. 1B). Also, these genes were mainly involved in sulfate assimilation pathway (Fig. 1C). The transcription of cysB did not change. Therefore, we speculated that SmpB deficiency was able to increase H_2_S synthesis.

### The production of H_2_S was increased in the absence of SmpB under nutritionally deficient conditions

**Figure 2:**
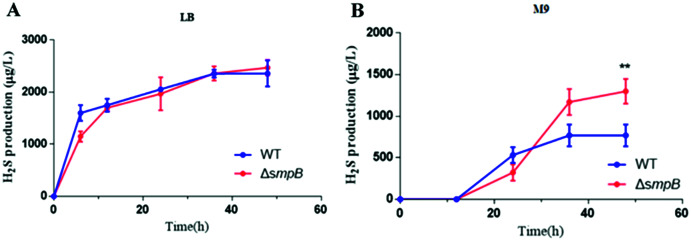
The production of H_2_S were increased in the absence of SmpB under nutritionally deficient conditions. (A) H_2_S production was measured by Pb(Ac)_2_-soaked paper strips in LB medium supplemented with five mg/L Na_2_SO_3_. No significant differences existed between WT and Δ*smpB* strains. (B) H_2_S production was measured by Pb(Ac)_2_-soaked paper strips in M9 medium supplemented with five mg/L Na_2_SO_3_. Values represented as means ± SD (*n* = 3). **p < 0.005 was determined by one-way ANOVA and Tukey post-test.

To figure out how sulfur metabolism was affected by SmpB deficiency, H_2_S production was measured in rich and deficient nutrition conditions by Pb(Ac)_2_ detection test. There is no difference between wild type (WT) and *smpB* deletion in a rich medium (LB medium) (Fig. 2A). Under the condition of nutritional deficiency (M9 medium), the *smpB* deletion produced less amount of H_2_S in the early stage of growth, but it enhanced to synthesize H_2_S in the stationary stage (Fig. 2B). The final H_2_S yield of *smpB* deletion was significantly higher than that of WT. This suggested that the production of H_2_S was increased in the absence of SmpB during auxotrophic conditions, especially predominant during the stationary phase of bacterial growth.

### Cbl affects the generation of H_2_S

**Figure 3:**
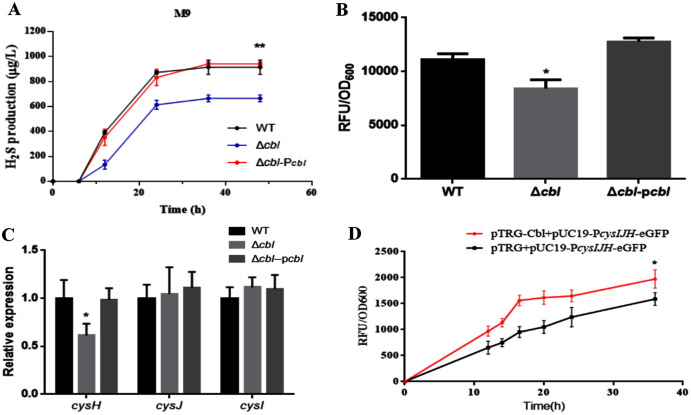
Cbl affected H_2_S production by promoting the transcription of *cysH*. (A) H_2_S production was measured by Pb(Ac)_2_-soaked paper strips in M9 medium supplemented with five mg/L Na_2_SO_3_. The tested strains included WT, Δ*cbl* and the complemented strain (Δ*cbl*-P*cbl*). Values represented as means ± SD (*n* = 3). **p < 0.005 was determined by one-way ANOVA and Tukey post-test. (B) Fluorescence intensities were detected by Synergy H1 (BioTek, Winooski, VT, USA) after the tested strains were treated with fluorescent H_2_S probe in M9 medium. Values represented as means ± SD (*n* = 3). *p < 0.01 was determined by one-way ANOVA and Tukey post-test. (C) The relative expressions of H_2_S synthesis genes were detected by qRT-PCR. Values represented as means ± SD (*n* = 3). *p < 0.01 was determined by one-way ANOVA and Tukey post-test. (D) Fluorescence intensities were detected by Synergy H1 (BioTek). The tested strains expressed P*cysIJH* only (pTRG+pUC19- P*cysIJH* -eGFP), and co-expressed both Cbl and P*cysIJH* (pTRG-Cbl+pUC19- P*cysIJH* -eGFP), respectively. Values represented means ± SD (*n* = 3). *p < 0.01 was determined by one-way ANOVA and Tukey post-test.

Using both the classic Pb(Ac)_2_ detection test and a fluorescent-based probe WSP5 (Zhang et al., 2020), we confirmed that, the production of H_2_S in *cbl* deletion strain was significantly lower than that of WT in M9 medium (Figs. 3A and 3B). And the difference was offset when Cbl protein was complemented (Figs. 3A and 3B). All the results were consistent with the transcriptome data, implying that Cbl had a positive regulatory effect on the synthesis of H_2_S under nutritional deficiency.

### Cbl promotes the transcription of *cysH*

The amino acid sequence of *cbl* gene was highly homologous to the *cysB* family, and CysB was proved to binding with the promoter of sulfur reductase (CysJIH) as a transcription factor for regulation. Therefore, it was speculated that *cbl* regulated the transcription of genes such as *cysH*, *cysJ* and *cysI*. The relative expression of *cysH* decreased significantly compared WT with cbl deletion by RT-qPCR, while those of *cysI*, *cysJ* revealed no differences (Fig. 3C).

Furthermore, the fusion of the promoter *cysJIH* (P*cysJIH*) and eGFP was constructed as the indicator plasmid for the fluorescent measurement. When co-expressed with Cbl, the fluorescence value was extremely significantly higher than that of the strain containing only P*cysJIH* (Fig. 3D). Collectively, Cbl had a positive regulation on P*cysJIH*.

### Cbl regulates downstream *cysH* by binding to the P *cysJIH*

**Figure 4:**
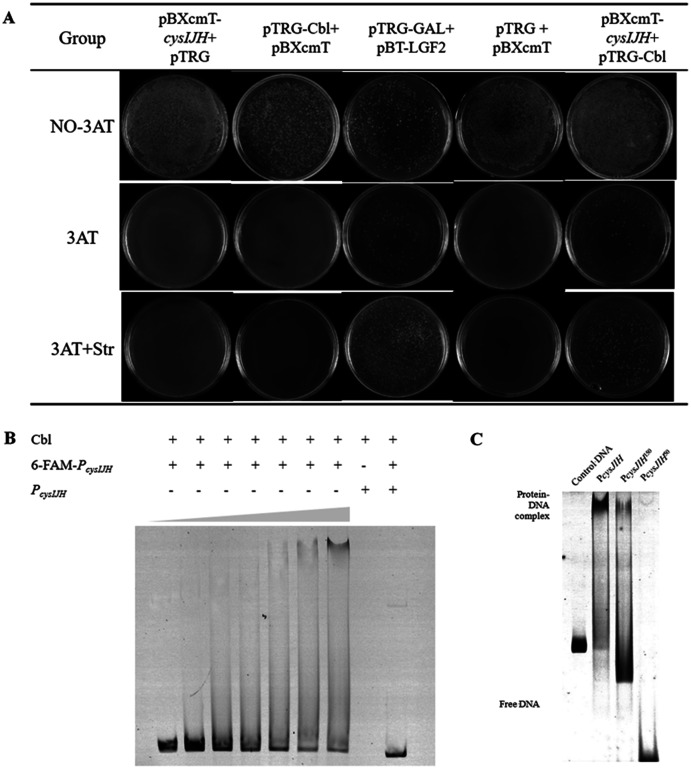
Cbl regulated downstream *cysH* by binding to the P*cysJIH*. (A) Results of bacterial one-hybrid. (B) Electrophoretic mobility shift assay (EMSA) for Cbl binding with P*cysJIH*. The 25 nM FAM-labeled P*cysJIH* was incubated with the increased amounts of Cbl protein. Cbl protein was titrated to the concentration of 0, 10, 20, 30, 40, 50 and 60 μM. (C) Electrophoretic mobility shift assay (EMSA) for Cbl binding with the varied size of P*cysJIH*. P*cysJIH* contained 226 bp upstream of transcriptional initiation site, P*cysJIH*^150^contained 150bp upstream of transcriptional initiation site, and P*cysJIH*^50^ contained 50 bp upstream of transcriptional initiation site. The 25 nM FAM-labeled probe DNA was incubated with 60 μM Cbl protein. The experiments were repeated in triplicate.

To confirm whether Cbl bound to P*cysJIH*, the P*cysJIH* promoter sequence and Cbl coding sequence were cloned into pBXcmT and pTGR plasmids respectively, and then co-transformed into *E. coli* XL 1-Blue MRF’ reporter strain for bacterial one-hybrid experiment. Only the co-expressed strain and the positive control grew on the minimum medium supplemented with six mM 3-AT and streptomycin (Fig. 4A), suggesting that the strong interaction between P*cysJIH* and Cbl.

Next, P*cysJIH* was labelled with Fluorophore 6-carboxy-fluorescein (6-FAM) for electrophoretic mobility shift assay (EMSA). The Cbl protein reduced the mobility of the 6-FAM-P*cysJIH* DNA probe corresponding to the increased Cbl concentration with the enhanced Cbl–DNA complex (Fig. 4B). So Cbl protein was able to bind with P*cysJIH* following with the regulation of H_2_S production.

### Determination of the binding region of P *cysJIH* with Cbl protein

To confirm the binding region of P*cysJIH* with Cbl protein, we truncated the full length of P*cysJIH* to 150 bp and 50 bp upstream of transcriptional initiation which were named as P*cysJIH*^150^ and P*cysJIH*^50^. P*cysJIH*^150^ was able to form a complex with Cbl protein, while P*cysJIH*^50^ lost the binding ability (Fig. 4C). The result suggested that the regions between 50 bp and 150 bp upstream of transcriptional initiation in P*cysJIH* were responsible for the binding of Cbl.

## Discussion

SmpB protein is involved in the regulation of multiple biological processes such as protein invasion, bacterial movement, central metabolism, lipopolysaccharide biosynthesis, two-component system, fatty acid metabolism, high temperature tolerance, cell cycle, and stress response (Shin & Price, 2007; Ansong et al., 2009; Barends et al., 2010). And the destruction of SmpB reduces the tolerance and adaptability of bacteria (Ansong et al., 2009).

Bacterial H_2_S has been proved toresist oxidative stress by reacting with reactive oxygen species (ROS), H_2_O_2_, etc. or stimulate catalase and superoxide dismutase to scavenging free radicals (Kimura, 2014; Mironov et al., 2017). Besides, the oxidative stress effect of H_2_S is also related to the defense of bacteria against antibiotics, because many antibiotics also trigger the production of ROS when they function as the targeted inhibition (Kohanski et al., 2007). So, the effect of H_2_S in scavenging ROS can make it more resistant to antibiotics.

In our study, the *smpB* deletion of *Aeromonas veronii* C4 was significantly higher in H_2_S production than wild type under M9 culture condition (Fig. 2B), implying that SmpB deficiency enhanced the H_2_S generation. Indeed, *smpB* deletion up-regulated multiple genes in the sulfate assimilation pathway, including *cysN, cysD*, *cysC*, *cysH*, *cysJ, cysI* and *cbl* (Figs. 1B and 1C).

In *Salmonella Typhimurium*, the promoter of *cysJIH* (P*cysJIH)* is regulated by CysB (Álvarez et al., 2015), which is homologous with Cbl (Kertesz, 2000). Therefore, we presumed that Cbl was responsible for the regulation of *cysH*, *cysJ* and *cysI* in *Aeromonas veronii* C4. Cbl bound to P*cysJIH* and positively regulated the transcription of *cysH* (Fig. 3D and Figs. 4A–4C).

Previously *smpB* deletion exhibits more tolerance to aminoglycosides antibiotic and oxidative stress under M9 culture (Fig. 2C) (Liu et al., 2018; Wang et al., 2019). In summary, we proposed that Cbl-regulated H_2_S generation compensated for the resistance and survival of SmpB damage under nutrient deficiencies, contributing to the adaptation and evolution of *Aeromonas veronii* against extreme environment.

## Conclusions

This study provided the first demonstration for the regulation between Cbl and *cysJIH*, and innovatively proposed the mechanism of Cbl-mediated H_2_S synthesis. Previously the strain of *smpB* deletion was observed to survive better than WT under the appropriate concentration of H_2_O_2_. In view of the function of H_2_S in oxidative resistance, we speculated that the accumulation of H_2_S increased the tolerance of oxidative resistance in SmpB deficiency. The results expanded the function of Cbl in pathogenic bacteria, and systematically explained the dynamic role of H_2_S in protecting bacteria from oxidative stress. These findings provide potential drug targets for aquatic diseases, offers theoretical basis for better understanding of bacterial pathogens resistance to environmental stress and supplies new ideas for clinical prevention and control of bacterial pathogens.

## Supplemental Information

10.7717/peerj.12058/supp-1 Supplemental Information 1 The information about the strains, plasmids and primers used in this study.

10.7717/peerj.12058/supp-2 Supplemental Information 2 RNA-sequencing of smpB-dependent gene expression in A. Veronii C4.

10.7717/peerj.12058/supp-3 Supplemental Information 3 Raw Data of qRT-PCR, H2S detection and Fluorescence detection.

## Additional Information and Declarations

### Data Availability

The following information was supplied regarding data availability: The raw measurements are available in the Supplemental File and at NCBI: PRJNA493739.
