# Supplementary material for: Cbl upregulates cysH for hydrogen sulfide production in Aeromonas veronii
Source: PeerJ. 2021 Sep 9;9:e12058. doi: 10.7717/peerj.12058 (PMC8435198; doi:10.7717/peerj.12058)
Supplement: Supplemental Information 1 [file peerj-09-12058-s001.docx]

Supplemental Table S1: Strains and plasmids used in this study.

| **Strain** | **Description** | **Sources** |
| --- | --- | --- |
| *Aeromonas veronii* C4 | Wild type, ampicillin resistance, virulent to fish. | Lab stock |
| *A. veronii* Δ*cbl* | Ampicillin resistance, deleted *cbl* gene from WT. | This study |
| *A. veronii* Δ*smpB* | Ampicillin resistance, deleted *smpB* gene from WT. | Lab stock |
| *A. veronii* Δ*cbl*-pBBR-*cbl* | Host strain for propagates pMD20-T and its derivatives. | Lab stock |
| *E.coli* WM3064 | Encoded a relaxase, a mating pair formation (MPF) complex and a type IV coupling protein in chromosome, diaminopimelic acid (Dap) auxotroph. | Lab stock |
| *E.coli* XL 1-Blue MRF’ reporter strain | Host strain for expressing pBXcmT, pTRG and their derivatives for one-hybrid systems, kanamycin resistances. | Lab stock |
| E.coli BL21 (DE3) | Host strain for protein expression. | Lab stock |
| **Plasmid** | **Description** | **Sources** |
| pRE112 | Chloramphenicol resistance, suicide plasmid for gene knock out, a conditional R6K ori which requiring the π protein for replication, expressed *sacB* for sucrose selection. | Lab stock |
| pRE112-*cbl* | Chloramphenicol resistance, pRE112 derivative for *cbl* knock out in A. veronii C4. | This study |
| pBBR-MCS-2 | Kanamycin resistance, broad-host-range cloning vector. | Lab stock |
| pBBR-*cbl* | Derivatives from pBBR-MCS-2, contains full ORF region of *cbl*. | This study |
| pTRG | Prey plasmid, ColE1 origin, tetracycline  resistance, *lpp/lac*-UV5 promoter. | Lab stock |
| pTRG-Cbl | Derivates from pTRG, expression transcriptional factor Cbl. | This study |
| pUC19 | *oriV* origin ampicillin resistance. | Lab stock |
| pUC-P*cysJIH*-eGFP | Ampicillin resistance, includes cysJIH promoter and expresses 9-residue at the N terminus of CysJ with full length eGFP | This study |
| pBXcmT | Ori1 origin, chloramphenicol resistance, includes *HIS3-aadA* reporter gene. | Lab stock |
| pBXcmT-P*cysJIH* | Derivates from pBXcmT, Chloramphenicol  resistance, P*cysJIH* was inserted in front of  *HIS3-aadA* reporter gene. | This study |
| pET-28a | Kanamycin resistance, T7 promoter, expresses His Tag. | Lab stock |
| pET-28a-Cbl | Derivates from pET-28a, expresses Cbl with  His Tag. | This study |
| pBT-LGF2 | Interaction control plasmid encoding the  dimerization domain (40 amino acids) of the  Gal4 transcriptional activator protein. | Lab stock |
| pTRG-Gal11 | Interaction control plasmid encoding a domain (90 amino acids) of the mutant form of the Gal11 protein. | Lab stock |

Supplemental Table S2: Primers used for qRT-PCR.

| **Primers** | **orward sequence(5'→3')** | **Reverse sequence(5'→3')** |
| --- | --- | --- |
| Av-*gyrB*-qF/R | GCTCACCATTCGTCGTAACGG | GCCACCCTCGTAGCAGAAAT |
| Av-*cysJ-*qF/R | CTGCTCAGGTGGTGGATGTGCT | TGACGGGGGTGTCGGGGTTTTG |
| Av-*cysI*-qF/R | CACTGTGGTGATCCCGCCCCAC | CGAAGCCGAAATCGCTTGCCTT |
| Av-*cysH*-qF/R | GAGCAGGGGGTAGAGGGGA | CACAGTGGGTGGTAGGGCA |
